# H3N2 Influenza Viruses with 12- or 16-Amino Acid Deletions in the Receptor-Binding Region of Their Hemagglutinin Protein

**DOI:** 10.1128/mBio.01512-21

**Published:** 2021-12-07

**Authors:** Huihui Kong, Shufang Fan, Kosuke Takada, Masaki Imai, Gabriele Neumann, Yoshihiro Kawaoka

**Affiliations:** a Influenza Research Institute, Department of Pathobiological Sciences, School of Veterinary Medicine, University of Wisconsin-Madison, Madison, Wisconsin, USA; b Division of Virology, Department of Microbiology and Immunology, Institute of Medical Science, University of Tokyo, Tokyo, Japan; c International Research Center for Infectious Diseases, Institute of Medical Science, University of Tokyo, Tokyo, Japan; National Institute of Allergy and Infectious Diseases, NIH; Washington University School of Medicine

**Keywords:** HA, hemagglutinin, influenza

## Abstract

Human influenza viruses evade host immune responses by accumulating mutations around the receptor-binding region of the hemagglutinin (HA) protein, which is composed of three key elements, the 130-loop, the 190-helix, and the 220-loop. Here, we characterized two human H3N2 influenza viruses with 12- and 16-amino acid deletions around the HA receptor-binding site that were isolated after antigenic selection of mutated H3N2 viruses. Structural modeling suggested that the 12-amino acid deletion eliminated the 190-helix. The 16-amino acid deletion comprises two stretches of 11- and 5-amino acid deletions. As the result of a frameshift, “novel” amino acids (not found in wild-type HA at these positions) are encoded between the deleted regions. Interestingly, structural modeling predicted that the novel sequence forms a structure resembling the 190-helix. However, compared to wild-type HA, the 16-amino acid deletion mutant lacks two antiparallel beta-sheets that connect the 190-helix and the 220-loop in wild-type HA. Nonetheless, both HA deletion mutants replicated in mammalian cells, and the 16-amino acid deletion mutant (with a remodeled 190-helix) also replicated in Syrian hamsters, albeit at low titers. Wild-type virus bound preferentially to α2,6-linked sialic acids, whereas both mutants gained affinity for α2,3-linked sialic acids. Moreover, the 12- and 16-amino acid deletions may affect the antigenic properties of the viruses. Thus, viruses with sizeable deletions around the HA receptor-binding site are viable but may display altered sialic acid preferences, altered antigenic properties, and attenuated replicative ability in cultured cells and virulence in Syrian hamsters.

## INTRODUCTION

Influenza A viruses, which belong to the *Orthomyxoviridae* family, consist of eight single-stranded negative-sense RNA segments, encoding 10 essential proteins and several accessory proteins. Based on the antigenic properties of the two viral envelope glycoproteins, hemagglutinin (HA) and neuraminidase (NA), influenza A viruses are divided into 18 HA subtypes and 11 NA subtypes. All subtypes have been isolated in avian species, except for the H17N10 and H18N11 subtypes, whose genetic material has been isolated from bats (1, 2).

Influenza viruses of the H1N1 and H3N2 subtypes are circulating in humans and cause frequent epidemics. Human influenza viruses are notable for their rapid evolution, and the annual reoccurrence of seasonal endemics or epidemics is attributed to frequent mutations in HA. The HA protein, which is the viral receptor-binding protein and the major target of the immune response, undergoes the fastest evolution among the viral proteins (3). By accumulating amino acid mutations in the major antigenic epitopes of HA, which are located close to the receptor-binding region (4–9), the virus evades neutralizing antibodies elicited in response to prior exposures to influenza viruses. These frequent changes are possible because of the high inherent tolerance for mutations at the antigenic sites of HA (10–13). Deletions in HA have been reported (14–18) but are relatively rare compared to amino acid changes and are typically not a major factor in the antigenic evolution of influenza viruses. However, a K134 deletion in an H1N1 virus was responsible for antigenic differences from other H1N1 viruses (16). Moreover, recent influenza B viruses of the Victoria lineage have acquired two- and three-amino acid deletions in their antigenic epitopes that have altered the antigenic properties of these viruses, which, in turn, required an update of the vaccine strain (17, 18).

Previously, we randomly mutated 17 amino acid positions in a human H3N2 HA protein that may affect antigenicity. Antigenic selection with human sera resulted in the isolation of several mutant viruses, including two with substantial amino acid deletions in the receptor-binding region. Here, we characterized these HA deletion mutants *in vitro* and *in vivo*.

## RESULTS

### Isolation and antigenic characterization of mutants with amino acid deletions in HA.

**FIG 1:**
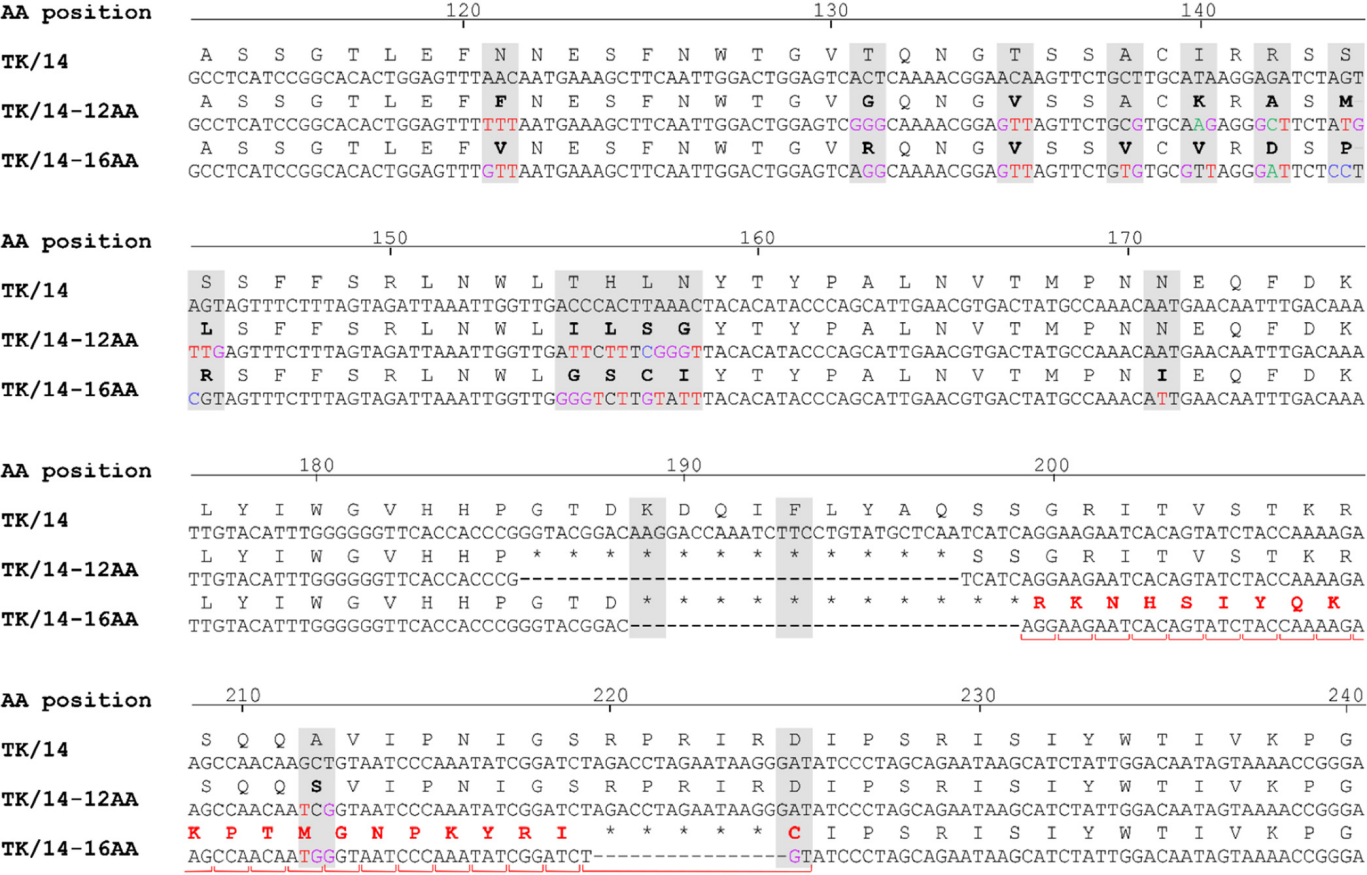
Sequence comparison of wild-type and mutant TK/14 HA proteins. Shown are the nucleotide and amino acid sequences of wild-type and mutant TK/14 HA proteins. The 17 amino acid positions targeted for mutagenesis are shaded in gray. The mutations at these positions detected in TK/14-12AA and TK/14-16AA are shown in boldface type. The amino acid deletions in TK/14-12AA and TK/14-16AA are indicated by asterisks. The novel amino acids generated in TK/14-16AA through a frameshift between the deleted regions are shown in red, bolded type.

The antigenicity of H3N2 human influenza viruses is affected by the amino acids close to the receptor-binding sites (4). Based on our previous work and published data, we selected 17 sites in HA (121, 131, 135, 138, 140, 142, 144, 145, 155 to 158, 171, 189, 193, 212, and 225; H3 numbering, used here) around the receptor-binding region to analyze the antigenicity of HA. Using a synthetic gene library that theoretically encodes all 20 amino acids at each of these 17 sites, we generated an A/Tokyo/UT-IMS2-1/2014 (H3N2, TK/14) virus library with up to 17 amino acid mutations at the selected sites of HA; the remaining viral segments were derived from TK/14 (NA segment) and from a high-yield A/PR8/34 (PR8-HY) virus (19). To isolate antigenic variants, the virus library was incubated with a mixture of 12 human sera (Table S1). A total of 63 viruses were isolated, and their HA segments were sequenced. Interestingly, we isolated two mutants with multiple amino acid deletions in HA. One mutant, TK/14-12AA, contained a 12-amino acid deletion encompassing amino acids 186 to 197 (Fig. 1); in addition, it encoded 12 amino acid changes at the targeted sites (Fig. 1). The other mutant, TK/14-16AA, lacked 16 amino acids (189 to 199 and 220 to 224) (Fig. 1); a frameshift between the deleted amino acid stretches also resulted in several novel amino acids not encoded by the wild-type HA protein (Fig. 1; novel amino acids created by the frameshift are shown in red, bolded type). TK/14-16AA also encoded 13 amino acid mutations at the targeted sites (Fig. 1).

10.1128/mBio.01512-21.1 TABLE S1 Microneutralization assays with human sera used for antigenic selection.^*^Titers are geometric means of two or three independent experiments. TK/14, A/Tokyo/UT-IMS2-1/2014 (H3N2); Maryland/14, recombinant A/Maryland/26/2014 (H3N2) virus; Alaska/15, recombinant A/Alaska/232/2015 (H3N2) virus; Nevada/16, recombinant A/Nevada/22/2016 (H3N2) virus; N/A, not applicable. Download Table S1, DOCX file, 0.02 MB. Copyright © 2021 Kong et al. 2021 Kong et al. https://creativecommons.org/licenses/by/4.0/ This content is distributed under the terms of the Creative Commons Attribution 4.0 International license.

To evaluate the antigenicity of the HA deletion variants, we first performed a focus reduction assay with ferret sera to human H3N2 viruses representing several antigenic clusters (Table 1). Interestingly, TK/14-12AA reacted with all of the sera tested, although the titers were reduced compared to the wild-type TK/14 virus titers. TK/14-16AA did not react with ferret sera generated to human H3N2 viruses that circulated from 2003 to 2009 but showed measurable focus reduction titers to ferret sera raised against human H3N2 viruses from 2011 to 2014 (Table 1). We also tested the HA deletion variants against the mixture of 12 human sera used for selection, as well as against the individual human sera (Table S1). The microneutralization titers of TK/14-12AA and TK/14-16AA against these sera were similar to those of wild-type TK/14, suggesting that properties other than antigenic differences may have contributed to their selection. These data indicate that viruses with sizable deletions in HA are viable and may be antigenically distinct from wild-type virus but do not completely lose their reactivity with human sera and ferret sera representing different antigenic clusters of human H3N2 viruses.

**TABLE 1:** Focus reduction neutralization titers of sera against the indicated viruses

Virus	Data for ferret serum:								
	A/Wyoming/3/2003	A/Wisconsin/67/2005	A/Brisbane/10/2007	A/Perth/16/2009	A/Victoria/361/2011	A/Texas/50/2012	A/Switzerland/9715293/2013	A/Hong Kong/4801/2014	TK/14^*a*^
A/Wyoming/3/2003	**22,567** ^*b*^	2,813	163	<20	<20	<20	36	66	108
A/Wisconsin/67/2005	657	**4,344**	1,456	41	72	73	<20	66	<20
A/Brisbane/10/2007	876	5,877	**5,618**	279	240	257	<20	428	<20
A/Perth/16/2009	<20	107	21	**899**	444	503	<20	122	102
A/Victoria/361/2011	93	108	121	477	**316**	454	88	363	76
A/Texas/50/2012	68	125	107	544	513	**671**	129	412	120
A/Switzerland/971529/3/2013	<20	<20	<20	73	198	499	**359**	1,266	81
A/Hong Kong/4801/2014	<20	90	<20	<20	87	159	206	**8,129**	200
TK/14	64	137	87	481	484	513	202	2,114	**432**
TK/14-12AA	106	61	66	71	103	166	138	394	52
TK/14-16AA	<20	<20	<20	<20	43	84	49	175	176

a TK/14, A/Tokyo/UT-IMS2-1/2014(H3N2).

b Homologous titers are shown in boldface type.

### Structure prediction of HA proteins with deletions.

**FIG 2:**
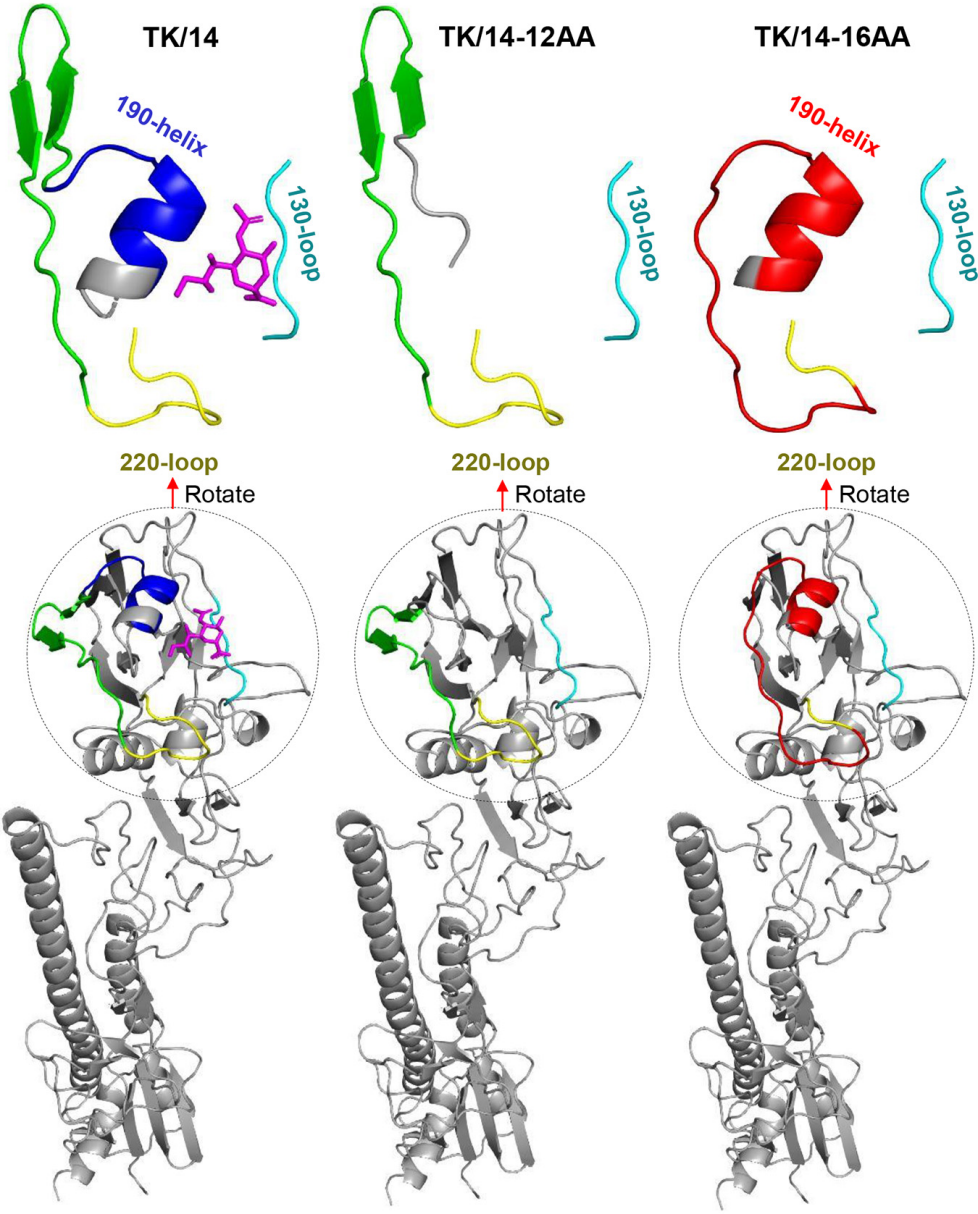
Prediction of the three-dimensional structure of the TK/14-12AA and TK/14-16AA HA proteins. The three-dimensional structure was predicted by using I-TASSER (21–23). Structural elements around the receptor-binding site are colored as follows: 130-loop, light blue; 190-helix, dark blue; 220-loop, yellow. The antiparallel β-sheets connecting the 190-helix and the 220-loop are shown in green. Sialic acid is shown in pink. The novel amino acids generated by the frameshift between the deleted regions are indicated in red.

The receptor-binding site of HA comprises three structural elements, the 130-loop (amino acids 134 to 138), the 190-helix (amino acids 190 to 198), and the 220-loop (amino acids 221 to 228) (20). To understand how the deletions in the TK/14-12AA and TK/14-16AA HA proteins affected these structural elements, we predicted the HA three-dimensional structure by using iterative threading assembly refinement (I-TASSER) (21–23). As shown in Fig. 2, the 12-amino acid deletion of TK/14-12AA resulted in the deletion of the 190-helix; however, the overall structure of the HA head region remained intact, consistent with our finding that the TK/14-12AA virus is viable and reacts with antisera to various human H3N2 influenza viruses. We expected that the two deletions in TK/14-16AA would eliminate the 190-helix and 220-loop; however, structural modeling predicted the formation of 190-helix- and 220-loop-like structures formed by the amino acids created by the frameshift between the two deleted amino acid stretches (Fig. 2; see also Fig. 1). Instead, the modeling predicted that TK/14-16AA would lack the two antiparallel β-sheets that connect the 190-helix and 220-loop in the wild-type HA protein (Fig. 2). Thus, despite a sizeable deletion in the HA head, the key structural elements of the HA receptor-binding region were maintained, yielding a viable virus.

### The NA protein is not required for the infectivity of TK/14 HA deletion mutants.

**FIG 3:**
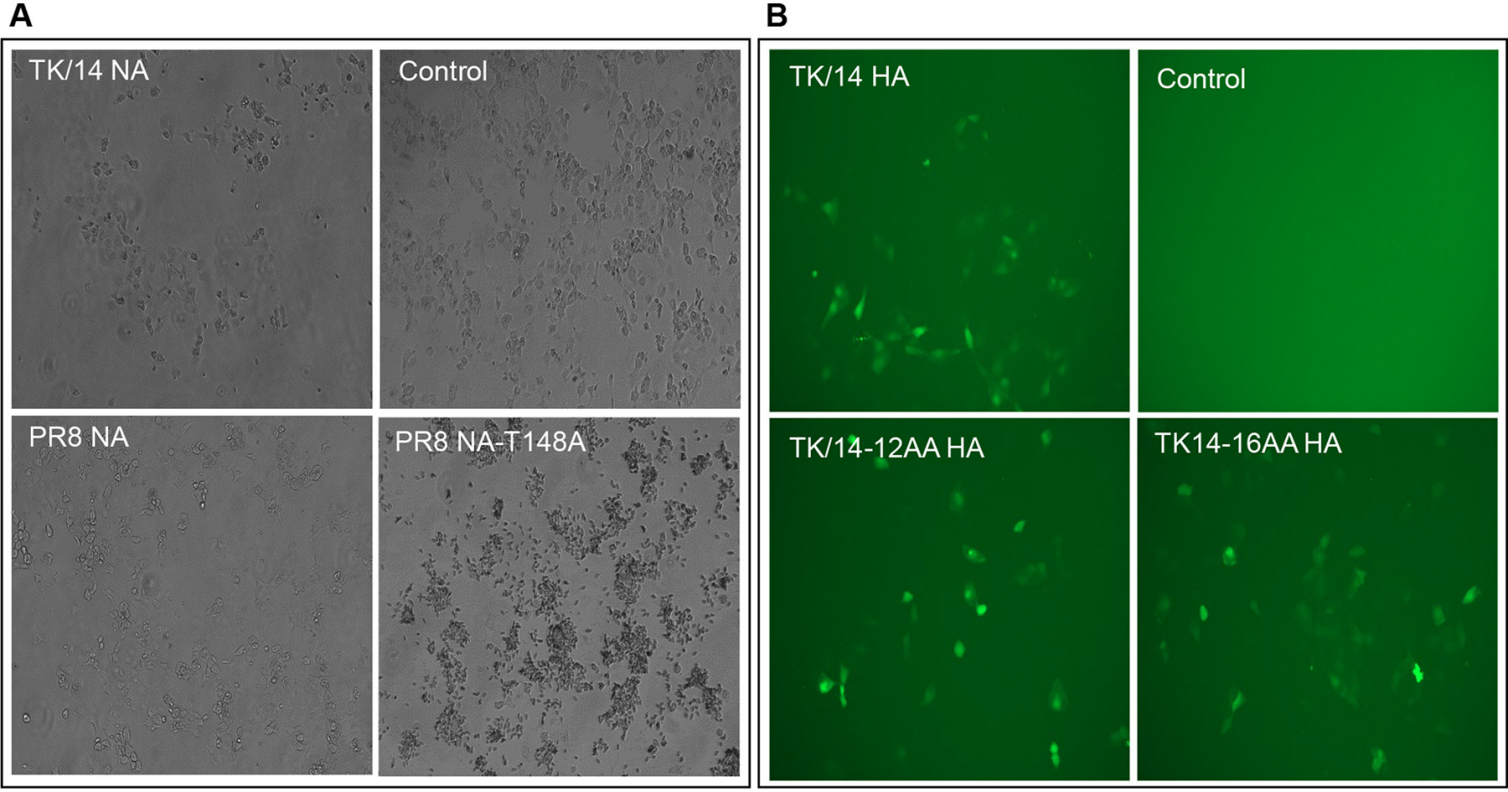
Hemadsorption and cell infection assays. (A) Hemadsorption assay. COS-1 cells were transfected with plasmids expressing the indicated NA protein; 48 h later, hemadsorption was measured by incubating the cells with 0.5% TRBCs for 30 min on ice. Hemadsorption was assessed under a light microscope. (B) Cell infection assay. Cell infectivity was measured by infecting AX4 cells with a virus in which the NA coding region was replaced with that of Venus; 48 h later, Venus expression was assessed at 488 nm. Control, no HA segment.

The HA of influenza viruses is the receptor-binding and fusion protein, whereas NA is a receptor-cleaving protein that releases viruses from the cell surface. However, since 2005, the NAs of human H3N2 viruses have also been shown to possess receptor-binding activity due to mutations such as T148K, H150R, and D151G (24–27). Therefore, for TK/14-12AA and TK/14-16AA, some of the biological functions of HA may be executed by NA. Sequencing analysis of the TK/14-12AA and TK/14-16AA NA proteins did not reveal any mutations. Nonetheless, we tested whether the TK/14-12AA and TK/14-16AA viruses used NA as a receptor-binding protein. First, we carried out hemadsorption assays by expressing NA proteins in eukaryotic cells and incubating the transfected cells with turkey red blood cells (TRBCs). Control PR8 NA protein did not bind to TRBCs (Fig. 3A), unlike PR8 NA-T148A, a laboratory-identified mutant NA protein known to agglutinate red blood cells. TK/14 NA did not bind to TRBCs, indicating that it likely does not serve as a receptor-binding protein for the TK/14-12AA and TK/14-16AA viruses.

To further test the potential contribution of NA to the receptor-binding of TK/14-12AA and TK/14-16AA, we performed cell infection assays with virus particles lacking NA. Cells were infected with a virus that contained all eight viral RNA (vRNA) segments; however, the coding region of NA was replaced with the Venus gene. Virus encoding wild-type TK/14 HA protein but lacking the NA protein was able to infect cells, resulting in Venus expression (Fig. 3B). Likewise, infection of cells with viruses possessing the TK/14-12AA or TK/14-16AA HA proteins but lacking the NA protein resulted in Venus expression, indicating that NA is not required for the entry of TK/14-12AA and TK/14-16AA viruses into cells.

### Receptor-binding preference of TK/14 HA deletion mutants.

**FIG 4:**
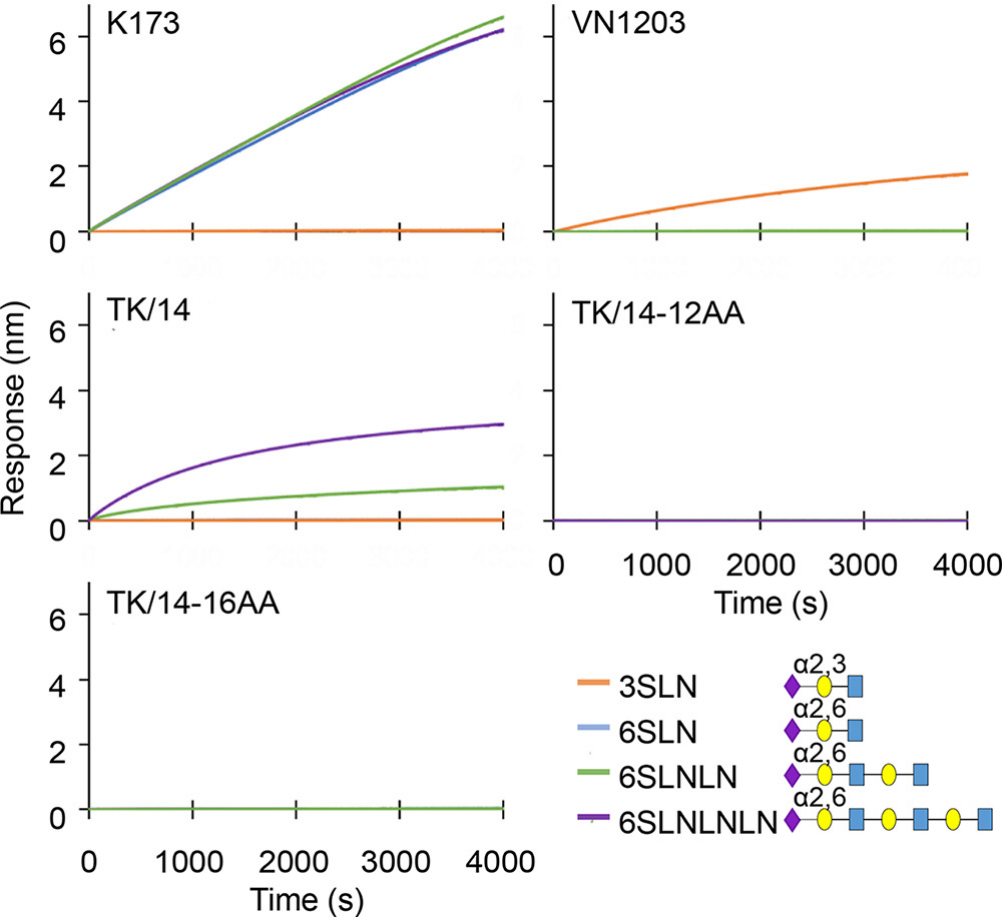
Receptor-binding preference. The receptor-binding preference of the indicated viruses was analyzed by use of BLI with synthesized receptor analogs. Four kinds of biotinylated glycans, 3SLN, 6SLN, 6SLNLN, and 6SLNLN, were immobilized to streptavidin biosensors. Then, the sensors were reacted with the purified virus for 4,000 s at 30°C. Blue square, *N*-acetylglucosamine; yellow circle, galactose; purple diamond, sialic acid. K173, A/Kawasaki/173/2001 (H1N1); VN1203, A/Vietnam/1203/2004 (H5N1); TK/14, A/Tokyo/UT-IMS2-1/2014 (H3N2).

Human influenza A viruses preferentially bind to α2,6-linked sialic acids, whereas avian influenza viruses preferentially bind to α2,3-linked sialic acids. To assess the sialic acid binding affinity of the TK/14 HA deletion mutants, we analyzed purified TK/14, TK/14-12AA, and TK/14-16AA viruses by using surface biolayer interferometry (BLI) with four different glycans (28–30). Human A/Kawasaki/173/2001 (H1N1; K173) and avian A/Vietnam/1203/2004 (H5N1; VN1203) control viruses bound to α2,6-linked and α2,3-linked sialic acids, respectively (Fig. 4). Wild-type TK/14 also bound to α2,6-linked sialic acids, albeit with weaker affinity than K173. In contrast, the TK/14-12AA and TK/14-16AA viruses displayed no detectable signal against the four receptor analogs tested.

To further characterize the receptor-binding properties of the TK/14 HA deletion mutants, we tested their binding to different types of red blood cells. Horse red blood cells (RBC), which exclusively express α2,3 sialosides (31), were used to test virus binding to α2,3-linked sialic acids. To measure virus binding to α2,6-linked sialic acids, TRBCs were treated with Vibrio cholerae neuraminidase and α2,6-(*N*)-sialyltransferase (32). The human K173 and avian VN1203 control viruses were used to confirm the specificity of the α2,3- and α2,6-expressing RBCs, respectively (Table 2). Wild-type human TK/14 virus also bound to α2,6 sialosides, as expected. Interestingly, we found that TK/14-12AA no longer bound to α2,6-expressing RBCs but gained the ability to bind to α2,3-expressing RBCs. TK/14-16AA displayed reduced binding to α2,6-expressing RBCs and gained the ability to bind to α2,3-expressing RBCs. These results indicate that the deletions in HA (partially) switched the TK/14 receptor-binding preference from human to avian type.

**TABLE 2:** HA titers of viruses against different types of red blood cell (RBC)

Virus^*a*^	Data for HA titer		
	TRBC	α2,6-sialic acid- expressing RBCs^*b*^	α2,3-sialic acid- expressing RBCs^*c*^
K173	5	4	0
VN1203	5	0	5
TK/14	5	4	0
TK/14-12AA	5	0	5
TK/14-16AA	5	2	4

a K173, A/Kawasaki/173/2001 (H1N1); VN1203); A/Vietnam/1203/2004 (H5N1); TK/14, A/Tokyo/UT-IMS2-1/2014 (H3N2).

b Turkey RBCs treated with *Vibrio cholera* neuraminidase and α2,6-(*N*)-sialyltransferase.

c Horse RBCs.

### Heat stability of TK/14 HA deletion mutants.

**FIG 5:**
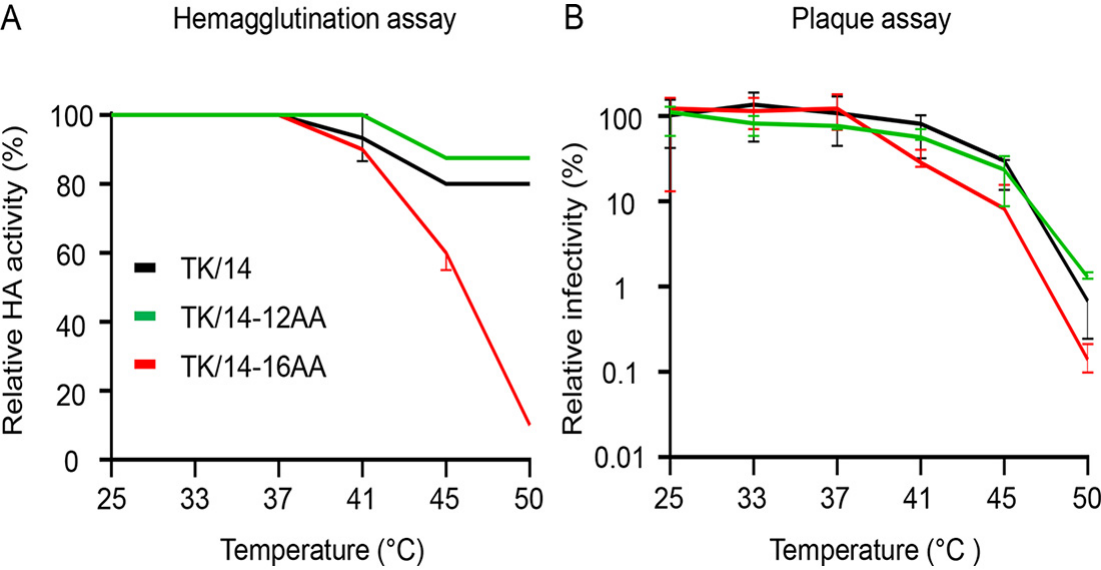
Heat stability of wild-type and mutant TK/14 viruses. (A and B) Viruses were incubated at the indicated temperature for 30 min, and then subjected to hemagglutination (A) and plaque (B) assays. The error bars represent the standard deviation of the mean of three independent experiments.

Substitutions in HA can affect its stability and the replication of influenza viruses (33, 34). We therefore tested the thermal stability of the HA deletion mutants by incubating them for 30 min at several temperatures from 25°C to 50°C and then performing HA and plaque assays (Fig. 5A and B). Compared with wild-type TK/14, the thermostability of TK/14-16AA was substantially reduced at temperatures above 37°C, but no appreciable differences were detected at the lower temperatures tested. In contrast, the thermostability of TK/14-12AA was only mildly reduced relative to that of wild-type TK/14 at the temperatures tested.

### Growth kinetics of TK/14 HA deletion mutants in eukaryotic cells.

**FIG 6:**
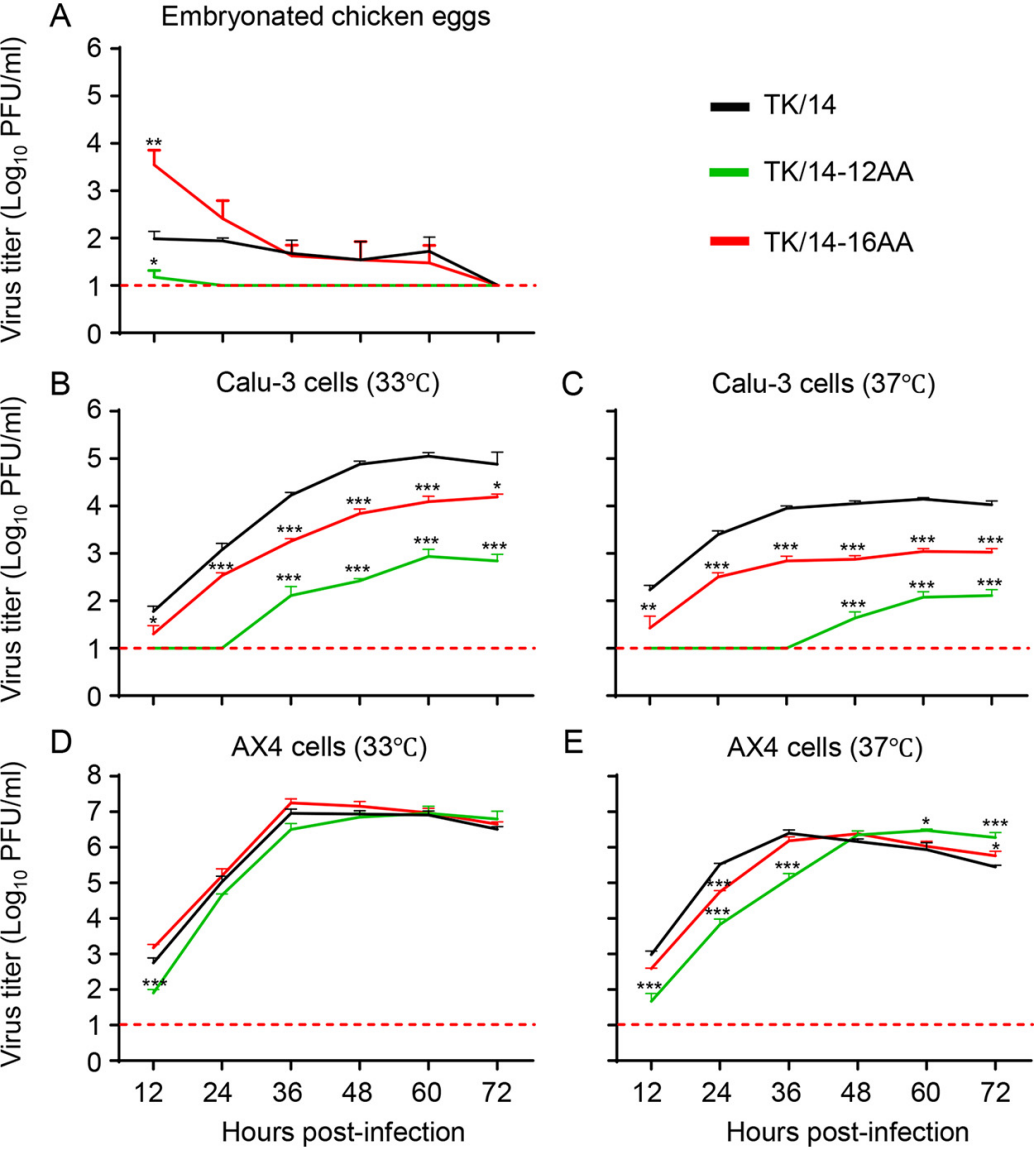
Growth kinetics of wild-type and mutant TK/14 viruses *in vitro*. (A to E) The growth efficiency of wild-type and mutant TK/14 viruses (with the remaining seven viral segments of TK/14) was evaluated in (A) embryonated chicken eggs, (B and C) Calu-3 cells, and (D and E) AX4 cells. Embryonated chicken eggs (four eggs per time point) were infected with 1 × 10^4^ PFU of the indicated viruses and incubated at 33°C. Calu-3 cells were infected at an MOI of 0.03 and incubated at (B) 33°C and (C) 37°C; AX4 cells were infected at an MOI of 0.001 and incubated at (D) 33°C and (E) 37°C. Allantoid fluid and cell culture supernatants were collected at 12-h intervals. Shown are the means from three independent experiments. Statistical analysis was carried out by use of a one-way ANOVA (B to E) or two-way ANOVA (A) followed by Dunnett’s *post hoc* test. Statistically significant differences between TK/14 and a mutant virus are indicated by asterisks. *, *P* < 0.05, **, *P* < 0.01, ***, *P* < 0.001.

Next, we evaluated the growth kinetics of TK/14, TK/14-12AA, and TK/14-16AA in embryonated chicken eggs and different mammalian cell lines; here, we used viruses that possessed all eight viral segments of TK/14 virus. In embryonated chicken eggs (Fig. 6A), virus titers, including those of wild-type TK/14, did not increase over time, indicating a lack of replication. TK/14-12AA and TK/14-16AA replicated in human lung epithelial Calu-3 cells at 33°C and 37°C, but their titers were significantly lower than those of TK/14 (Fig. 6B and C). Comparison of the two HA deletion mutants showed that TK/14-12AA (which lacks the 190-helix) was more severely attenuated than TK/14-16AA (which lacks the antiparallel β-sheets that connect the 190-helix and the 220-loop). Since the two HA deletion mutants were isolated in AX4 cells, which is an MDCK cell line with increased levels of α2,6-linked sialic acid (35), we evaluated their growth efficiency in this cell line as well. In AX4 cells, TK/14-12AA and TK/14-16AA replicated to relatively high titers at 33°C and 37°C (Fig. 6D and E), although both mutants were mildly (TK/14-12AA) to moderately (TK/14-16AA) attenuated compared with the wild-type virus at early time points.

### Replication of TK/14 HA deletion mutants in Syrian hamsters.

**FIG 7:**
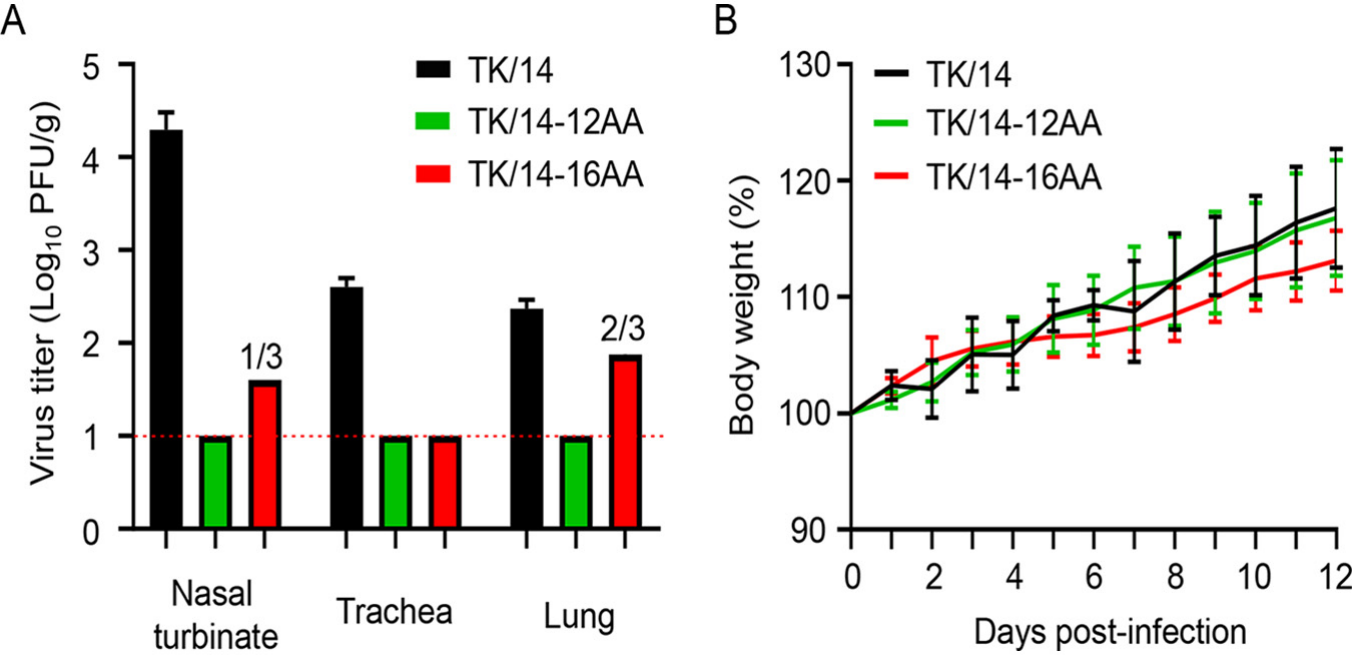
Virus replication in Syrian hamsters. (A) Virus replication and (B) weight changes of wild-type- and mutant TK/14 virus-infected Syrian hamsters. Six hamsters were intranasally inoculated with 10^6^ PFU of the indicated virus in a volume of 100 μL. Three hamsters were euthanized on day 3 postinoculation, and the nasal turbinates, trachea, and lungs were collected for virus titration in AX4 cells. The other three hamsters were monitored for weight changes for 2 weeks. Data represent the means and standard deviations of results obtained from each hamster.

Our data to this point indicated that TK/14-12AA and TK/14-16AA are viable but attenuated *in vitro* compared with wild-type TK/14 virus. To test the replication of these viruses *in vivo*, we inoculated Syrian hamsters with 10^6^ PFU of TK/14, TK/14-12AA, or TK/14-16AA (all possessing the remaining seven viral segments of wild-type TK/14). Three hamsters were euthanized on day 3 postinfection for virus titration, whereas the remaining three hamsters were monitored for 2 weeks for body weight changes. As expected, wild-type TK/14 replicated in the lungs, trachea, and nasal turbinates of infected Syrian hamsters (Fig. 7A). TK/14-12AA was not isolated from the nasal turbinates, trachea, or lungs on day 3 postinfection (Fig. 7A), demonstrating a lack of detectable replication in Syrian hamsters. TK/14-16AA replicated to low titers in the nasal turbinates and lungs of hamsters (Fig. 7A), but virus replication was detected in only one or two of the three animals, respectively. Moreover, the body weights of the infected hamsters increased steadily during the observation period (Fig. 7B), demonstrating the low pathogenicity of these viruses to Syrian hamsters.

## DISCUSSION

Here, we isolated two human H3N2 viruses with amino acid deletions in HA, one with a 12-amino acid deletion and the other with a 16-amino acid deletion. These deletions did not abolish the receptor-binding function of HA but switched the receptor-binding preference from human toward avian type. The mutants replicated relatively efficiently in mammalian cells, and the 16-amino acid-deletion mutant also replicated in the respiratory organs of Syrian hamsters, albeit inefficiently. These data demonstrate that H3 HA can accommodate deletions around the receptor-binding site, although the viruses may be attenuated and are, therefore, unlikely to emerge in nature and circulate among humans.

Our structural modeling of the TK/14-16AA deletion mutant suggested a refolding of HA such that the deleted 190-helix and 220-loop were recreated by unrelated amino acids resulting from a frameshift between the two deleted amino acid stretches (Fig. 1). This finding demonstrates the extraordinary plasticity of HA in which unrelated amino acids appear to form a structure very similar to the wild-type structure. However, as a result of these rearrangements, the TK/14-16AA HA lacks the antiparallel β-sheets that connect the 190-helix and 220-loop. In contrast, structural modeling predicted the deletion of the 190-helix in TK/14-12AA, resulting in a viable, but highly attenuated, virus. Overall, deletion of the 190-helix (in TK/14-12AA) had a stronger attenuating effect than the replacement of the original 190-helix with a similar structure composed of different amino acids (TK/14-16AA). Interestingly, similar deletions in HA have been described for two other influenza viruses (14, 15); incubation of human H3N2 X-31 virus with monoclonal antibodies resulted in the selection of an antigenic escape variant with a deletion of amino acids 224 to 230 (14). Based on the pH dependence of membrane fusion, the deletion mutant was less stable than the wild type, consistent with our finding that the TK/14-16AA deletion mutant was less thermostable than wild-type TK/14 virus. Moreover, an eight-amino acid deletion at positions 221 to 228 of HA emerged in North American H7N2 viruses in 1996 and has become stable in this lineage (15). Crystallographic analysis of a virus from this lineage showed that deletion of the 220-loop had limited structural impact on the 190-helix and 130-loop (36). Together, these and our findings indicate that influenza viruses can accommodate sizeable deletions in the receptor-binding region, but these deletions may lead to attenuation.

The human K173 and TK/14 viruses interacted with α2,6 sialosides that are commonly used to measure the receptor-binding specificity of influenza viruses (Fig. 4). In contrast, the TK/14-12AA and TK/14-16AA deletion mutants did not display detectable interaction with these sialosides, demonstrating that their sialic acid specificity differed from that of the wild-type virus. However, the TK/14-12AA and TK/14-16AA deletion mutants maintained their binding to TRBCs and gained the ability to bind to α2,3-sialic acid-expressing RBCs; this binding profile was paralleled by undetectable (TK/14-12AA) or reduced (TK/14-16AA) ability to bind to α2,6-sialic acid-expressing RBCs (Table 2). Hence, the deletions in HA changed the receptor-binding specificity from human toward avian type. Interestingly, changes in receptor-binding specificity were also described for the X-31 and H7 deletion mutants described earlier. Comparable to the TK/14-12AA and -16AA deletion mutants, the X-31 mutant (with a deletion of amino acids 224 to 230) gained the ability to bind to α2,3-sialic acid (14). Compared with older North American H7N2 viruses, the naturally emerged H7N2 viruses with the HA 221 to 228 deletion maintained their preference for α2,3-sialic acids and acquired limited binding to α2,6-sialic acids (36). Collectively, these data demonstrate that deletions around the receptor-binding site may not be deleterious but can change the sialic acid specificity of HA.

The deletions in TK/14-12AA and TK/14-16AA reduced the focus reduction titers against ferret sera to several human H3N2 viruses from 2003 to 2014 but did not completely eliminate their reactivity with these ferret sera. TK/14-12AA (which lacks the 190-helix) maintained reactivity with all sera tested here. TK/14-16AA (with 190-helix- and 220-loop-like structures formed by amino acids not found in TK/14) interacted with sera from 2011 on, but not with older sera. Moreover, both deletion variants reacted with the human sera used for selection comparably to wild-type virus (Table S1), suggesting that antigenic escape may not have played a major role in the selection of TK/14-12AA and TK/14-16AA. Instead, the efficient replication of TK/14-12AA and TK/14-16AA in AX4 cells (Fig. 6) suggest that efficient binding to these cells may have been important in the isolation of TK/14-12AA and TK/14-16AA.

In summary, our findings expand our knowledge of HA plasticity by demonstrating that sizeable deletions can be accommodated but may lead to attenuation and altered receptor-binding specificity and antigenic properties.

## MATERIALS AND METHODS

### Viruses and cells.

293T human embryonic cells and COS-1 African green monkey kidney fibroblast cells were purchased from the American Type Culture Collection (ATCC, USA) and maintained in Dulbecco’s modified Eagle medium (DMEM) containing 10% fetal bovine serum (FBS). Polarized human bronchial epithelial Calu-3 cells from ATCC were grown in MEM supplemented with 5% newborn calf serum DMEM/nutrient mixture F-12 containing 10% FBS. AX4 cells, generated in our laboratory, were cultured in MEM supplemented with 5% newborn calf serum and maintained in MEM containing 0.3% bovine serum albumin (MEM/BSA) (35). A/Tokyo/UT-IMS2-1/2014 (H3N2, TK/14) was isolated in Tokyo, Japan, and maintained in our laboratory.

### Virus generation.

Before generating the virus library, an HA plasmid library theoretically containing all 20 amino acids at 17 sites in HA (121, 131, 135, 138, 140, 142, 144, 145, 155 to 158, 171, 189, 193, 212, and 225) was generated as previously described (13). To generate the virus library, the HA plasmid library was cotransfected into 293T cells with seven RNA polymerase I-controlled plasmids synthesizing the NA gene of TK/14 and the six internal genes of a high-yield A/PR8/34 (PR8-HY) virus (19), four plasmids expressing the viral replication complex (PB2, PB1, PA, and NP), and one plasmid expressing human airway trypsin-like protease (HAT) (37). The 293T supernatant was collected 48 h posttransfection, incubated with a mixture of 12 human sera, and subjected to plaque assays in AX4 cells. The HA genes of 63 virus plaques were sequenced, resulting in the isolation of TK/14-12AA and TK/14-16AA. To analyze the growth kinetics and replication of the wild-type and mutant viruses *in vitro* and *in vivo*, TK/14, TK/14-12AA, and TK/14-16AA were generated with the internal genes of TK/14. Briefly, 293T cells were cotransfected with the respective HA plasmid, the remaining genes of TK/14, and five plasmids expressing PB2, PB1, PA, NP, and HAT. The 293T supernatant was collected and amplified in AX4 cells. Recombinant A/Maryland/26/2014, A/Alaska/232/2015, and A/Nevada/22/2016 viruses (possessing the HA and NA genes of the respective virus with the remaining genes of A/Puerto Rico/8/34 virus) were generated by using reverse genetics (37). All virus stocks were sequenced before use.

### Focus reduction neutralization assay.

The focus reduction assay (FRA) was performed in 96-well cell plates by using the protocol described previously (38). AX4 cells were used for the FRA in this study. First, the number of focus-forming units (FFU) was determined by immunostaining. Briefly, we prepared a 10^0.5^-fold diluted series of virus in MEM/BSA, and then 50 μL of the diluted virus was added to wells containing 50 μL of MEM/BSA. After a 2-h incubation at 37°C, 100 μL of 0.6% Avicel (FMC, Ireland) in MEM/BSA containing 0.5 μg/mL TPCK (tosylsulfonyl phenylalanyl chloromethyl ketone)-trypsin was added to each well and cultured for 16 h. The cell plate was then fixed with 4% formalin, washed with 0.1% Tween 80 in phosphate-buffered saline (PBST), and incubated with 5% Triton X-100 in PBS at room temperature for 20 min. After incubating with a mouse anti-NP monoclonal antibody pool (International Reagent Resource, USA) and goat anti-mouse IgG antibody (SeraCare, USA), a KPL TrueBlue kit (SeraCare) was used for visualization. The FFU titer was counted by using an AID ISPOT robot (AID, USA).

To determine the serum titer, 50 μL of 2-fold serially diluted serum was added to the AX4 cell plate, and then 400 FFU of the indicated virus in 50 μL was added to the serum-covered cells. After incubating the plate for 2 h at 37°C, 100 μL of 0.6% Avicel containing 0.5 μg/mL TPCK-trypsin was added, and the plate was cultured for another 16 h. Finally, the FFU in each well was quantified as described above. The focus reduction neutralization titer is the reciprocal dilution of serum corresponding to 50% foci reduction calculated by generating nonlinear regression curves using Prism 8 software (GraphPad, USA).

### Microneutralization assay.

The microneutralization assay is a serologic test to detect the presence of antibodies that prevent virus infectivity. First, the serum was diluted in a series of 2-fold dilutions in MEM/BSA medium containing 2 μg/mL TPCK-trypsin. Second, 50 μL of the serially diluted serum was mixed with 50 μL of MEM/BSA containing 100 PFU of the indicated virus and then incubated at 37°C for 1 h. The mixture was then transferred onto AX4 cells in 96-well plates, and the cells were cultured at 33°C for 3 days. The microneutralization titer is the highest dilution that inhibits the formation of a cytopathic effect.

### Hemadsorption assay.

The NA proteins of the indicated viruses were expressed in COS-1 cells by transfecting the cells with pCAGGS-NA plasmids using Lipofectamine 2000 (Thermo Fisher Scientific, USA). The medium was replaced with fresh cell culture growth medium 24 h posttransfection, and the cells were cultured for another 24 h. Then, the cells were washed with cold DMEM and incubated with cold 0.5% turkey red blood cells (TRBCs) on ice. After half an hour of incubation, the cells were washed with cold DMEM five times and observed under a light microscope.

### Cell infection assay.

To test whether the TK/14-12AA and TK/16AA HA proteins mediate cell entry, influenza viruses were generated that express only HA protein on the viral surface, with the NA coding region being replaced with that of the reporter protein Venus. Briefly, we constructed an NA gene composed of the 3′ noncoding region of the PR8 NA viral RNA segment, 183 bases corresponding to the 5′-terminal coding sequence of NA (maintained because these sequences contain the NA vRNA packaging signal [39]; the NA start coding was disabled), the Venus cDNA, 157 bases corresponding to the 3′-terminal coding sequence of NA (maintained because these sequences contain the NA vRNA packaging signal [39]), and the 5′ noncoding region of the PR8 NA viral RNA segment. Viruses with wild-type or mutant HA segments (or without an HA segment) were generated in the presence of neuraminidase from Clostridium perfringens (Sigma, USA) by using reverse genetics (37). The 293T supernatant was collected 48 h posttransfection and used to infect AX4 cells; 48 h later, the cells were observed at 488 nm.

### Biolayer interferometry.

The receptor-binding preference of the indicated viruses was evaluated by using biolayer interferometry (FortéBio, USA) with biotinylated glycans as previously described (40, 41). First, viruses were inactivated with β-propiolactone and purified by 30% sucrose density ultracentrifugation. Then, the viruses were quantified by using a bicinchoninic acid (BCA) protein assay kit (Pierce, USA). For biolayer interferometry, we used 50 pM of virus in HBS-EP (150 mM NaCl, 10 mM HEPES [pH 7.4], 3 mM EDTA, 0.005% surfactant P20). Four biotinylated glycans, Neu5Ac(α2-3)Gal(β1-4)GlcNAc (3SLN), Neu5Ac(α2-6)Gal(β1-4)GlcNAc (6SLN), Neu5Ac(α2-6)Gal(β1-4)GlcNAc(β1-3)Gal(β1-4)GlcNAc (6SLNLN), and Neu5Ac(α2-6)Gal(β1-4)GlcNAc(β1-3)Gal(β1-4)GlcNAc(β1-3)Gal(β1-4)GlcNAc (6SLNLNLN)], were purchased from Tokyo Chemical Industry. To immobilize the glycans to the streptavidin biosensors (FortéBio, USA), the glycans were diluted to HBS-EP and then reacted with the biosensors. Binding was measured for 4,000 s at 30°C. The data were collected, processed, and analyzed by using Octet Data Analysis software.

### Hemagglutination assays with normal or resialylated red blood cells.

Hemagglutination assays were performed by adding 50 μL of 0.5% RBCs to 50 μL of 2-fold serially diluted virus in V-bottom 96-well plates. The mixture was incubated for 30 min at room temperature. The hemagglutination titer is the highest dilution that agglutinates RBCs.

To generate TRBCs expressing only α2,6-linked sialic acids, 100 μL of a 1% solution of TRBCs in phosphate-buffered saline (PBS) was treated with 50 mU of Vibrio cholerae neuraminidase (Roche, USA) in 8 mM CaCl_2_ at 37°C for 2 h. The treated TRBCs were resuspended with PBS containing 1% BSA after washing three times with PBS. Removal of sialic acids was confirmed by the complete loss of agglutination activity of the control viruses. Then, 180 μL of Vibrio cholerae neuraminidase-treated TRBCs were incubated with 2 mU of α2,6-N-sialyltransferase (Sigma, USA) and 1.5 mM CMP-sialic acid (Sigma, USA) at 37°C for 2 h. After being washed three times with PBS, the RBCs were diluted to 0.5% with PBS containing 1% BSA for further use.

### HA heat stability.

The thermostability of wild-type and mutant TK/14 viruses was analyzed at different temperatures. Briefly, a PCR tube (Applied Biosystems, USA) containing the same amount of the indicated virus in 50 μL of MEM/BSA was incubated for 30 min at 25°C, 33°C, 37°C, 41°C, 45°C, and 50°C and then quickly cooled to 4°C. The titers of all aliquots were tested by use of a hemagglutination assay with 0.5% TRBCs and by performing plaque assays in AX4 cells. The experiments were performed in triplicate.

### Evaluation of growth efficiency.

The growth efficiency of the wild-type and mutant viruses (possessing internal genes from wild-type TK/14) was evaluated in 10-day-old embryonated chicken eggs, AX4 cells, and Calu-3 cells. The embryonated eggs (four eggs per time point) were injected with 10^4^ PFU of the indicated virus and cultured at 33°C for the indicated times. Allantoic fluids were collected, and virus titers were assessed by performing plaque assays in AX4 cells. AX4 and Calu-3 cells were infected with the indicated viruses at a multiplicity of infection (MOI) of 0.001 and 0.03, respectively. Briefly, the cells were washed with PBS twice, infected with the indicated viruses for 1 h, and again washed twice. The cells were maintained at 33°C and 37°C in MEM/BSA containing 0.5 μg/mL TPCK-trypsin. Supernatants were collected at 12-h intervals and titrated in AX4 cells by conducting plaque assays at 33°C. The experiments were conducted in triplicate.

### Virus replication in Syrian hamsters.

Male Syrian hamsters (4 weeks old; ENVIGO, USA) were used in this study. Per virus, six hamsters were intranasally inoculated with 10^6^ PFU of the indicated virus (possessing the seven remaining viral segments from wild-type TK/14) in a volume of 100 μL. Three Syrian hamsters were euthanized on day 3 postinfection (p.i.), and the nasal turbinates, trachea, and lungs were collected for virus titration; the other three hamsters were weighed daily for 2 weeks. Then, 3 weeks postinfection, the animals were euthanized; the blood was collected and used for focus reduction and microneutralization assays.

### Statistical analysis.

Statistical analyses were performed by using Prism 8 software. Raw data from growth curves were converted to the logarithmical scale before further analysis. One-way analysis of variance (ANOVA) followed by Dunnett’s *post hoc* test was used to assess differences in viral growth curves in culture cells. Two-way analysis of variance (ANOVA) followed by Dunnett’s *post hoc* test was used to compare viral titers in embryonated eggs. A *P* value of less than 0.05 was considered significant.
