# Supplementary material for: H3N2 Influenza Viruses with 12- or 16-Amino Acid Deletions in the Receptor-Binding Region of Their Hemagglutinin Protein
Source: mBio. 2021 Dec 7;12(6):e01512-21. doi: 10.1128/mBio.01512-21 (PMC8649756; doi:10.1128/mBio.01512-21)
Supplement: TABLE S1 [file mbio.01512-21-st001.docx]

**Table S1. Microneutralization assays with human sera used for antigenic selection**

| Serum | Collection date | Donor  age | Year of birth | Neutralizing titer* | | | | | |
| --- | --- | --- | --- | --- | --- | --- | --- | --- | --- |
|  |  |  |  | Maryland/14 | Alaska/15 | Nevada/16 | TK/14 | TK/14-12AA | TK/14-16AA |
| LQ236255 | 4/8/2015 | 28 | 1987 | 33 | <20 | 20 | 25 | 63 | 40 |
| LQ236231 | 4/8/2015 | 29 | 1986 | 80 | 57 | 40 | 80 | 127 | 63 |
| LQ236244 | 4/8/2015 | 31 | 1984 | 40 | 28 | 20 | 20 | 50 | 20 |
| LQ236263 | 4/8/2015 | 32 | 1983 | 226 | 80 | 40 | 50 | 254 | 63 |
| LQ236220 | 4/8/2015 | 52 | 1963 | 160 | 80 | 40 | 142 | 160 | 80 |
| LQ236266 | 4/8/2015 | 54 | 1961 | 24 | 28 | ≤20 | 20 | 63 | 20 |
| LQ236226 | 4/8/2015 | 58 | 1957 | 57 | 57 | 80 | 101 | 226 | 127 |
| LS8826189A | 7/25/2016 | 19 | 1997 | 80 | 80 | 40 | 160 | 127 | 160 |
| LS5553405A | 7/27/2016 | 22 | 1994 | 269 | 160 | 40 | 160 | 89 | 160 |
| LS2385911A | 7/27/2016 | 30 | 1986 | 134 | 57 | 40 | 50 | 320 | 20 |
| LS8826222A | 7/26/2016 | 35 | 1981 | 190 | 113 | 160 | 40 | 71 | 20 |
| LS8826185A | 7/25/2016 | 39 | 1977 | 80 | 40 | 80 | 80 | 101 | 50 |
| Mixed Sera | N/A | N/A | N/A | 113 | 80 | 67 | 101 | 127 | 80 |

*Titers are geometric means of two or three independent experiments.

# TK/14, A/Tokyo/UT-IMS2-1/2014 (H3N2); Maryland/14, recombinant A/Maryland/26/2014 (H3N2) virus; Alaska/15, recombinant A/Alaska/232/2015 (H3N2) virus; Nevada/16, recombinant A/Nevada/22/2016 (H3N2) virus; N/A, not applicable.
